# Genome-wide association studies of inflammatory bowel disease in German shepherd dogs

**DOI:** 10.1371/journal.pone.0200685

**Published:** 2018-07-20

**Authors:** Atiyeh Peiravan, Francesca Bertolini, Max F. Rothschild, Kenneth W. Simpson, Albert E. Jergens, Karin Allenspach, Dirk Werling

**Affiliations:** 1 Department of Pathology and Pathogen Biology, Royal Veterinary College, University of London, North Mymms, United Kingdom; 2 Department of Animal Science, Iowa State University, Ames, Iowa, United States of America; 3 College of Veterinary Medicine, Cornell University, Ithaca, NY, United States of America; 4 College of Veterinary Medicine, Iowa State University, Ames, Iowa, United States of America; University Hospital Llandough, UNITED KINGDOM

## Abstract

Canine Inflammatory Bowel Disease (IBD) is considered a multifactorial disease caused by complex interactions between the intestinal immune system, intestinal microbiota and environmental factors in genetically susceptible individuals. Although IBD can affect any breed, German shepherd dogs (GSD) in the UK are at increased risk of developing the disease. Based on previous evidence, the aim of the present study was to identify single nucleotide polymorphisms (SNPs), which may confer genetic susceptibility or resistance to IBD using a genome-wide association study (GWAS). Genomic DNA was extracted from EDTA blood or saliva samples of 96 cases and 98 controls. Genotyping of cases and controls was performed on the Canine Illumina HD SNP array and data generated was analyzed using PLINK. Several SNPs and regions on chromosomes 7,9,11 and 13 were detected to be associated with IBD using different SNP-by-SNP association methods and F_ST_ windows approach. Searching one Mb up-and down-stream of the most significant SNPs, as identified by single SNP analysis as well as 200Kb before and after the start and the end position of the associated regions identified by F_ST_ windows approach, we identified 63 genes. Using a combination of pathways analysis and a list of genes that have been reported to be involved in human IBD, we identified 16 candidate genes potentially associated with IBD in GSD.

## 1. Introduction

Inflammatory bowel disease (IBD) is a chronic debilitating disease that affects both humans and dogs. In dogs, it represents a group of common chronic enteropathies characterized by persistent or recurrent gastrointestinal signs (GI) and with histological evidence of inflammation (usually lymphoplasmacytic and/or eosinophilic) in the lamina propria of the small intestine, large intestine or both [1]. Despite recent progress and improvement in the diagnosis of IBD in dogs, the treatment of this condition remains challenging. IBD is not a curable disease, therefore the aim of current treatment protocols is to minimise the severity and frequency of the clinical signs including vomiting and diarrhoea. In general, treatment protocols include sequential dietary [2, 3], antibiotic [4,5] and corticosteroid [6] treatment trials. Currently, treatment with anti-inflammatory medications such as corticosteroid is the mainstay treatment for both human IBD and canine IBD patients [4]. However, the response to treatment is highly variable, and up to 50% of dogs with IBD that are initially managed with steroids will develop resistance and/or significant side effects, which ultimately leads to euthanasia in many dogs [6–8]. Recent advances in the field of molecular genetics in human IBD make it possible to use the patients’ genetic profile to predict the response to treatment [9, 10]. Similar to human, understanding the genes involved in canine IBD is predicted to reveal insights into disease pathogenesis in canine IBD. This could lead to the development of genetic screening tests for diagnosis and a “personalized medicine” approach, where dogs will be treated with specifically targeted therapeutics, taking their individual genetic makeup into account.

Although IBD can affect any dog, breed-specific associations and disease phenotypes have been reported in the literature [11, 12]. In the United Kingdom (UK), German shepherd dogs are at increased risk of developing the disease [13]. The exact aetiology of IBD in humans as well as dogs is not well understood, although it is considered to be a multifactorial immune-mediated disease in both species, resulting from a complex interaction between the intestinal innate and adaptive immune systems, the intestinal microbiome, and the genetic make-up of a potentially susceptible individual [14–17].

While recent genome-wide association studies (GWAS), investigating the complex genetic basis of IBD in humans have resulted in the identification of 163 susceptibility loci [18], the genetic factors contributing to susceptibility to canine IBD remain largely unknown. Genetic studies into canine IBD, using a candidate gene approach, have revealed a breed-independent association with non-synonymous single nucleotide polymorphisms (SNPs) in Toll-like receptor (TLR)-5, a gene encoding an important mucosal pattern recognition receptor (PRR). In addition, breed-specific SNPs in TLR-4 and nucleotide binding oligomerization domain protein 2 (NOD-2) were identified in GSD [19–21]. A recent study also reported association between SNPs in Major histocompatibility class (MHC) II haplotypes and a potentially increased resistance to IBD in GSD [22]. Despite of these findings, it is believed that similar to human IBD, canine IBD is a complex polygenic disorder with involvement of several more genetic factors that remain to be identified.

The development of high-throughput SNP genotyping technologies has facilitated the detection of genes responsible for complex diseases of the dog. Furthermore, long linkage disequilibrium within breeds resulting from genetic bottlenecks during domestication of dogs and breed formation, made the dog population structure suitable for association mapping techniques [23,24].

The aim of the current study was to detect additional genetic factors associated with IBD in a defined GSD population using GWAS approach. SNP profiles for a total of 190 GSDs were generated using the Canine Illumina HD SNP array. Using the resulting data set, GWAS was performed, which resulted in the subsequent identification of additional loci associated with IBD in GSD, some of them also having been described for human IBD.

## 2. Materials and methods

### 2.1. Ethics and welfare statement

All blood samples used in this study were collected in ethylenediaminetetraacetic acid (EDTA) and were residual material following completion of diagnostic testing, used for research with informed written owner consent. The use of residual EDTA blood and buccal swab samples was approved by the RVC Ethics and Welfare Committee (reference number 2013 1210).

### 2.2. Study population and genotyping

The case population consisted of 96 GSDs based in the UK, which were diagnosed with IBD at either the Royal Veterinary College (University of London, Uk), the Small Animal Teaching Hospital (University of Cambridge, UK), the Animal Health Trust (Newmarket, UK), or three small animal referral centres including Anderson Moores Veterinary Specialists (Hampshire, UK), Davies Veterinary Specialists (Hitchin, UK) and Hope Vets (Bovingdon, UK). The case population was identified using stringent inclusion criteria: all cases have been affected by chronic gastrointestinal signs (i.e. > 3 weeks duration), including diarrhoea and/or vomiting and/or anorexia and/or weight loss. Other causes of chronic gastrointestinal disease had been excluded, by performing routine haematology, serum biochemistry, faecal parasitology and bacteriology, abdominal ultrasonography, serum ACTH stimulation tests and measurement of serum canine trypsin‑like immunoreactivity concentration (TLI), as described in detail previously [22]. All dogs had undergone gastrointestinal endoscopic examination for the collection of mucosal intestinal biopsies. Histological examination of the biopsies had revealed lymphoplasmacytic and/or eosinophilic infiltration of the lamina propria. None of the cases suffered from concurrent diseases of known or suspected immune-mediated origin, dermatological conditions or diseases for which GSD are known to be predisposed for (i.e. anal furunculosis, atopic dermatitis or superficial keratitis).

The control population included 98 GSDs based in the UK. Animals in this group were only considered for inclusion in the study if they were older than 8 years of age, therefore reducing the likelihood to develop IBD later in life. Control dogs were retrospectively phenotyped, either by owner or veterinary surgeon telephone contact, to ensure they had neither current nor previous history of gastrointestinal signs for a duration of more than 5 consecutive days and did not suffer from conditions of known or suspected immune-mediated origin.

Residual blood samples stored in EDTA anticoagulant were available from the RVC sample archive for a total of 69 GSD diagnosed with IBD and 81 breed-matched geriatric controls. EDTA blood samples were also available for 10 cases that have been seen at the University of Cambridge (8 cases) and the Animal Health Trust (2 cases). In addition, buccal swab samples from the other cases and controls for which a residual blood sample was not available were provided by owners for inclusion in the control population.

Genomic DNA (gDNA) was extracted from blood using the GenElute™ Blood Genomic DNA Kit (Sigma-Aldrich^®^, Gillingham, UK) and from saliva samples using the Performagene^®^ Kit (DNA Genotek Inc, Canada) according to manufacturer’s instructions. Following extraction, the amount of gDNA in all samples were quantified by nucleic acid fluorescent staining using a commercial kit and following the manufacturer’s instructions (Quant-iT™ PicoGreen® dsDNA Kit, Life Technologies, Paisley, UK), samples were normalised to a concentration of 20 ng μl^-1^ and stored at -20°C until use.

The genotyping of all cases and controls was performed using the Illumina 170K CanineHD® Beadchip (Illumina, San Diego, CA, USA). All SNPs with call rate <90%, minor allele frequency < 2%, monomorphic, unmapped to the CanFam3.1 reference sequence (GCF_000002285.3) and samples with call rate <80% were removed. After quality control, 94 cases, 96 controls and 117K SNPs remained for the final analysis.

To assess presence of population stratification, identity-by-decent and multidimensional scaling (MDS) analyses were performed using PLINK v1.07 software [25]. A scatter plot for the first 2 dimensions was produced. Population stratification analysis was also performed on the dataset with the Admixture software [26], with number of subpopulations (K) that ranging from 1 to 7. The cross-validation error generated at each K value was used to determine the best number of subpopulations across the dataset that is represented by the K with the lowest cross validation error.

### 2.3. Genome-wide association study

Association analyses were performed using different approaches: a basic case and control association (chi-square allelic test), a logistic regression and a logistic regression using the C1 and C2 components previously calculated with the MDS analysis as covariate in PLINK v1.07 software. P = -log10(5e-08) was considered as significant association, P = -log10(1e-05) considered as suggestive association. Genomic inflation factor (λ) and quantile-quantile (Q–Q) plots (S1 Fig) were obtained using the GenABEL software [27].

### 2.4. Fixation index (Fst) analysis

Association analysis was also performed using F_ST_ analysis SNP by SNP and using a sliding window of 1Mb, with 500Kb of overlapping. F_ST_ was calculated SNP by SNP, using the adapted formula below [23]

$$Fst_{k}=N_{k}/D_{k}$$

Where k is the SNP marker *k*, with frequency *p*_*1*_^*[k]*^, *p*_*2*_^*[k]*^

$$N_{k}=p_{1}{}^{\left[k\right]}(q_{2}{}^{\left[k\right]}-q_{1}{}^{\left[k\right]})+p_{2}{}^{\left[k\right]}(q_{1}{}^{\left[k\right]}-q_{2}{}^{\left[k\right]})$$

$$D_{k}=p_{1}{}^{\left[k\right]}q_{2}{}^{\left[k\right]}+q_{1}{}^{\left[k\right]}p_{2}{}^{\left[k\right]}=N_{k}+p_{1}{}^{\left[k\right]}q_{1}{}^{\left[k\right]}+p_{2}{}^{\left[k\right]}q_{2}{}^{\left[k\right]}$$

The values can range from 0 (no differences) to 1 (complete different). For the window-based analysis, the mean F_ST_ was calculated across the SNPs included in the window.

The top four SNPs for single SNP and 5 windows for overlapping windows were considered as the most divergent between case and control groups.

### 2.5. Identification of potential candidate genes

Gene searching was conducted with a range of ±1 Mb for the SNPs based analyses and ±200Kb for the regions identified with the windows based analysis. To identify potential candidate genes associated with IBD in GSDs, pathway analysis was performed using the Enrichr tool, (http://amp.pharm.mssm.edu/Enrichr) to detect genes involved in pathway that could be related with the disease. For this analysis, the default statistical tests and corrections for multiple testing to maintain an overall p-values of 0.05 were applied. Moreover, all the genes identified were compared to a list of genes that has been already stablished for human IBD [18], to search for common genes. Genes that were detected with each of these two approaches were considered for the discussion.

## 3. Results

**Fig 1:**
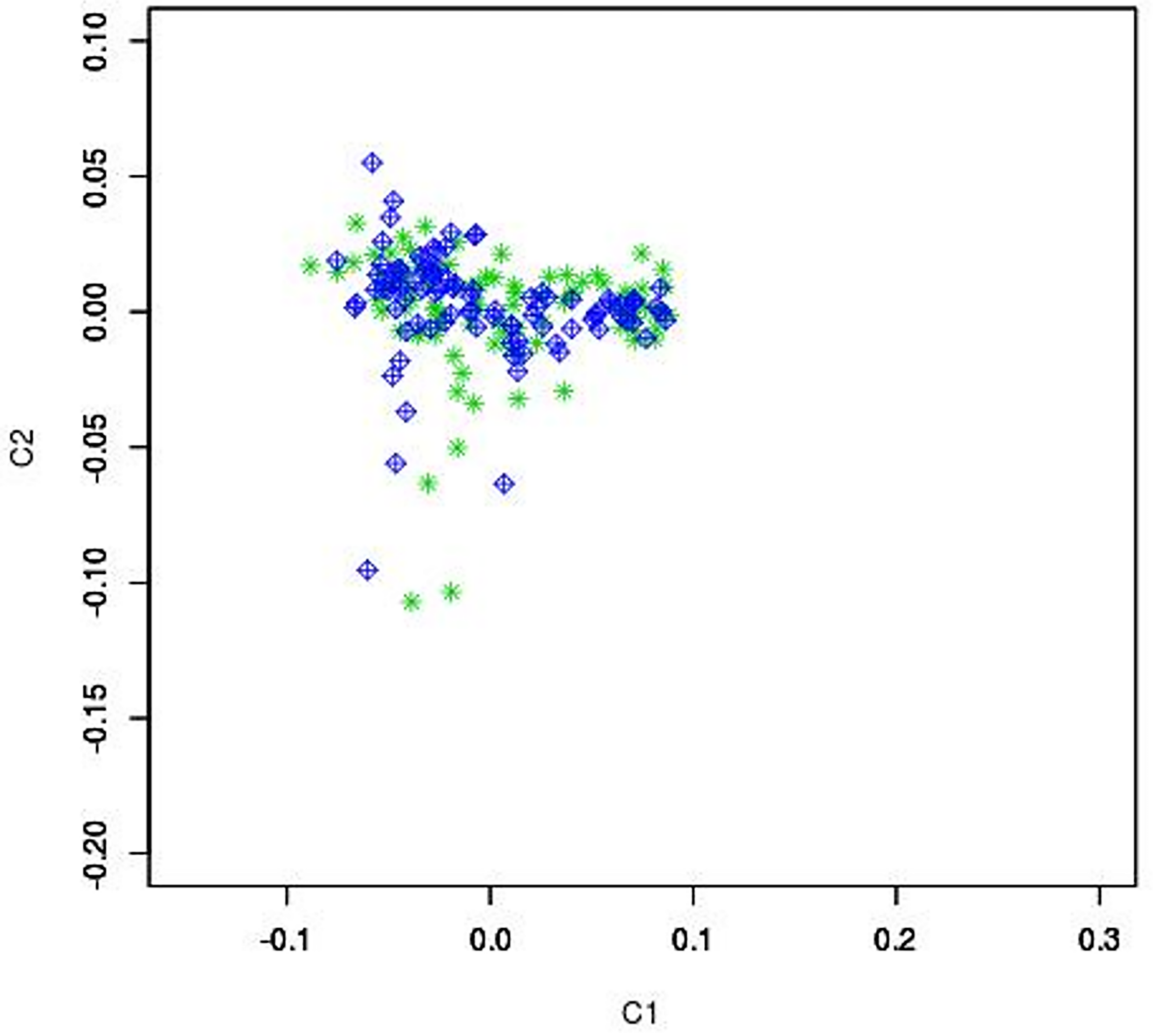
Scatter plot for the first 2 dimensions. The x-axis is principal component 1 and y-axis is principal component 2. Green: case, Blue: control.

**Fig 2:**
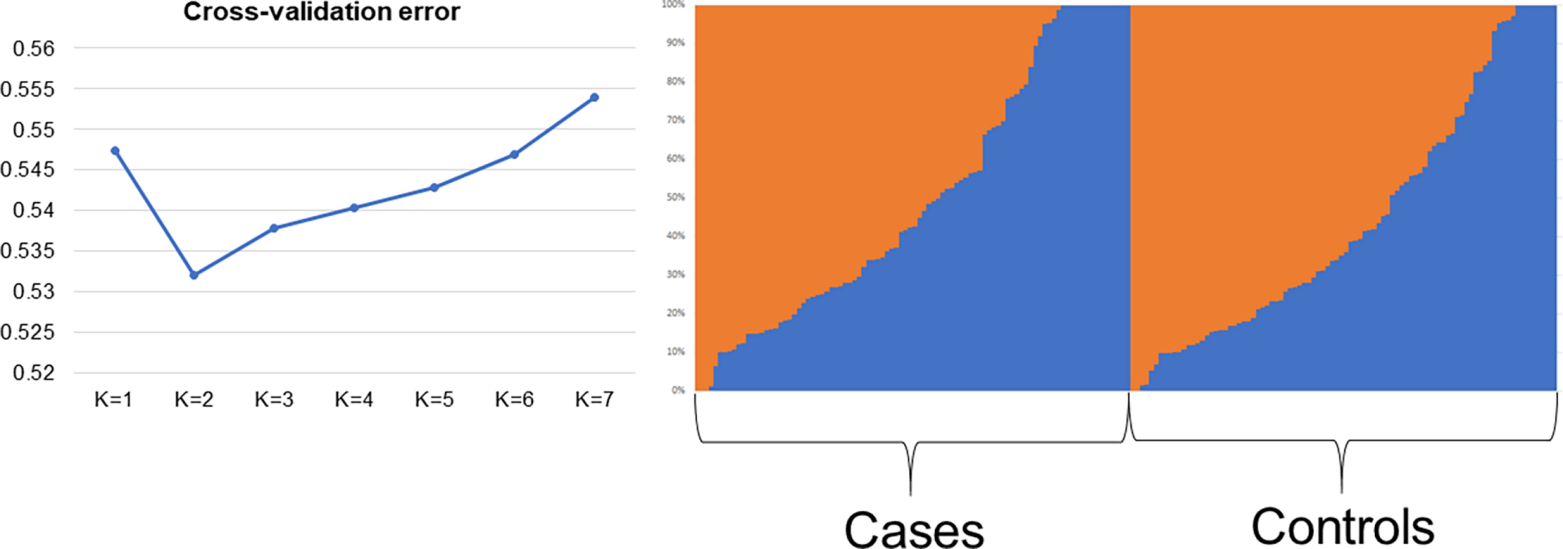
**Admixture plot of the case-control dataset (showed on the right) using the K with the lowest cross-validation error (showed on the left)**.

This study is the first GWAS on GSDs with the aim of investigating genetic factors associated with IBD. We performed genotyping of 170,000 SNP markers of the entire GSD cohort. The exclusion of non-informative markers, markers with low call rate and individuals with call rates <80% resulted in 200 individuals (94 cases, 96 controls) and 117K markers passing QC. Principal component analysis with PLINK (Fig 1) showed no evidence for population stratification within our GSD cohort (n = 190). The admixture analysis showed only two subpopulation that are balanced between cases and controls (Fig 2).

Results of the identity-by-descend analysis revealed three pairs of individuals with high Identity-by-descent proportion values (P_HAT> 0.90) in our study population. One sample of each pair was removed (two were cases and one control) prior to association analysis, resulting in 92 cases and 95 controls.

### 3.1. Association tests

**Fig 3:**
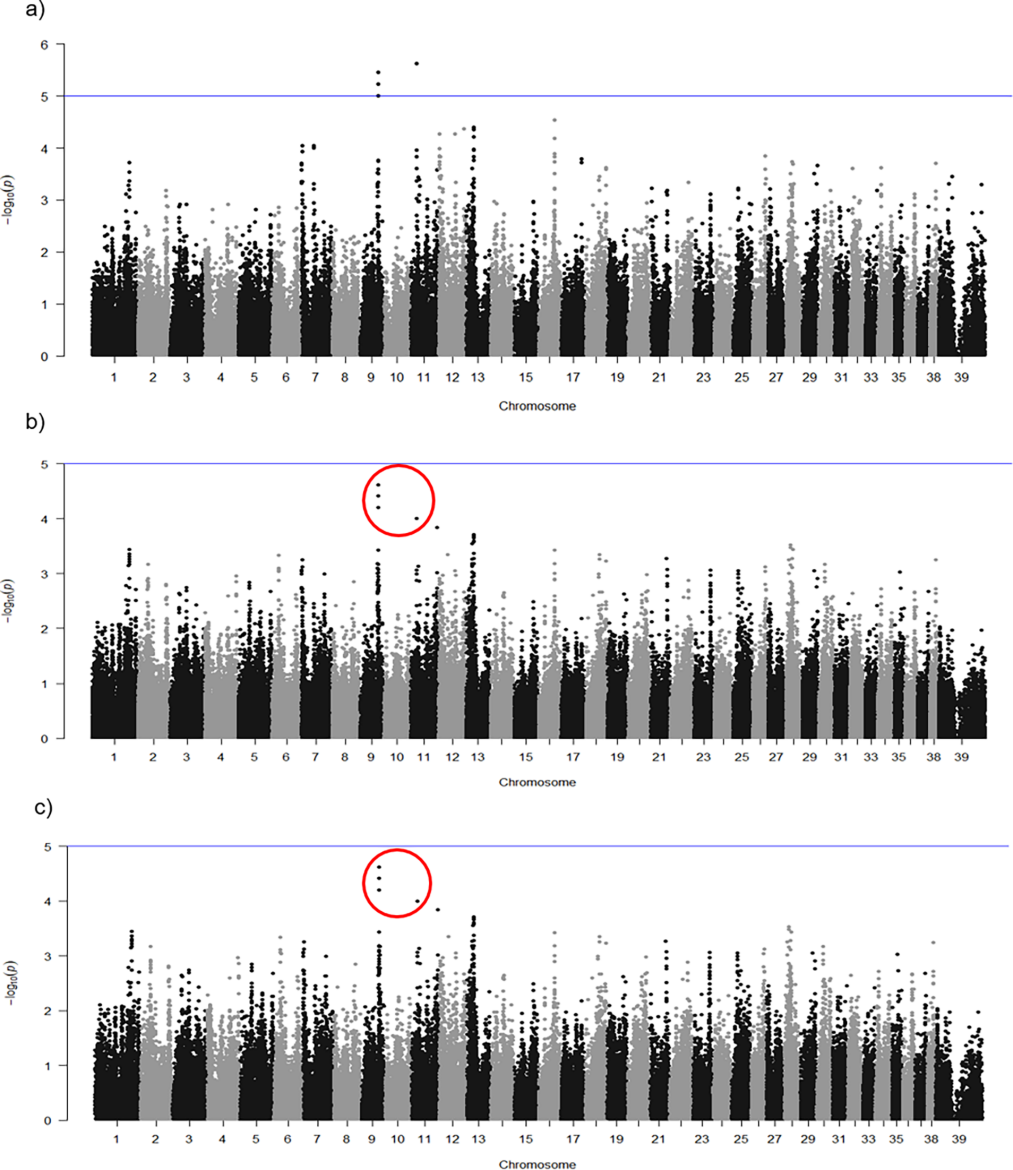
**Manhattan plots of basic case-control association test (a), logistic regression without covariate (b) and logistic regression with covariate (c).** Suggestive association threshold is indicated with the blue line. SNPs of the logistic analyses with the lowest p-value that overlapped the basic association study are within red circles.

IBD in GSD appears to be associated with SNPs on Chromosomes 11. GWAS of 92 cases versus 95 controls (total of 187 GSDs), using different statistical approaches, identified a number of SNPs with high differences on chromosomes 7, 9, 11 and 13. Basic case/control association analysis (Fig 3A) revealed three SNPs on chromosome 9 and one SNP on chromosome 11 with suggestive association. The strongest association (P = 2.36 × 10^−6^) identified by basic case/control association analysis was for SNP BICF2S23033111 at position 20,056,580 on chromosome 11. A second peak was within a region on chromosome 9 spanning from position 51,531,181 to 51,544,743 bp. In this region, three SNPS (BICF2P812982, BICF2P436494 and BICF2P753594) were identified, Results are shown in Table 1.

**Table 1:** The most significant SNPs found by basic case/control association analysis.

CHR	SNP	BP	A1	F_A	F_U	A2	CHISQ	P	OR
9	BICF2P753594	51531181	C	0,07609	0,2447	G	19,54	9,86E-06	0,2542
9	BICF2P436494	51541093	G	0,07609	0,25	A	20,52	5,91E-06	0,2471
9	BICF2P812982	51544743	G	0,07609	0,2553	A	21,51	3,52E-06	0,2402
11	BICF2S23033111	20056580	C	0,212	0,04787	A	22,28	2,36E-06	5,349

CHR: Chromosome

SNP: SNP ID

BP: Physical position on CanFam3.1

A1: Minor allele name (based on whole sample)

F A: Frequency of this allele in cases

F U: Frequency of this allele in controls

A2: Major allele name

CHISQ: Basic allelic test chi-square

P: Asymptotic p-value for this test

OR:Estimated odds ratio (for A1, i.e. A2 is reference)

Logistic association approaches were also performed as our trait of interest was binary. These SNPs were found to have the lowest p-values by both logistic associations with no covariate (Fig 3B and S1 Table) and with covariate (Fig 3C and S2 Table), although they did not reach the significance threshold (Fig 3B and 3C and S1 and S2 Tables respectively).

Quantile–quantile plots for the three analyses (S1 Fig) showed the relationship between observed (y-axis) and expected (x-axis) test statistics and visualize the population substructure. The slight deviation in the upper right tail from the y = x line are suggestive of associations. Data generally falling on the y = x lines suggests no clear systemic bias. The degree of deviation from this line is measured by the 𝜆-statistic, where a value close to 1 indicates that the data follows the normal chi-squared distribution. The logistic with no covariate (Figure B in S1 Fig) showed the lowest 𝜆, which can be considered as most conservative model. The correction with population derived covariate (Figure C in S1 Fig) showed a difference in values. However, in both three cases, big differences were not observed, as the study population is fairly homogenous [28].

### 3.2. Fixation index (Fst) tests

**Fig 4:**
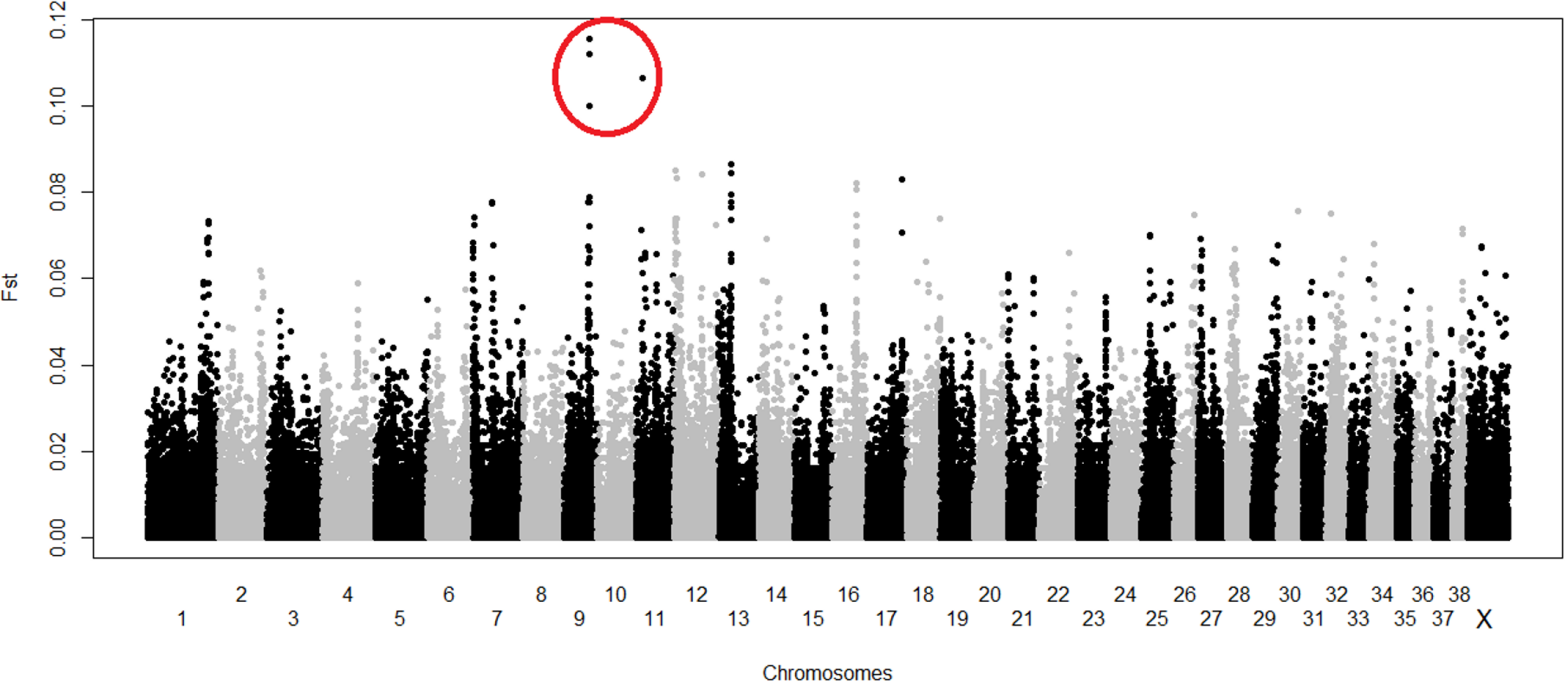
Single F_ST_ approach result. SNPs with the highest F_ST_ value that overlap the basic case/control association test and logistic association are within red circles.

The F_ST_ analyses was carried out SNP by SNP, reported the top 5 most divergent SNPs on chromosomes 9, 11 and 12 (Fig 4 and S3 Table). Anyway, the to 4 SNPs, three on chromosome 9 and 1 on chromosome 11, overlapped those previously identified with the association analyses.

The results of the Fst window-based analysis indicated five main divergent regions between cases and controls: four on chromosome 7 and one on chromosome 13 with the highest m F_ST_ value (Table 2).

**Table 2:** Regions on chromosome 7 and 13, found by F_ST_ windows approach.

chromosome	Start	End	N.Snps	mF_ST_
7	4000000	5000000	31	0.0362679
7	3500000	4500000	44	0.0384714
13	2500000	3500000	33	0.038702
7	2500000	3500000	17	0.0403834
7	3000000	4000000	39	0.0408544

Start: start position of the window. End: End position of the window. N.Snps: number of SNPs included in each window. mF_ST_: mean F_ST_ value calculated for SNPs included in each window.

### 3.2 Candidate genes

The gene searching of the SNPs and windows-based analysis identified 80 genes, 44 detected with considering the consensus SNPs of the singles SNPs analyses (S4 Table), and 36 considering the regions detected with the windows-based Fst analysis (S5 Table). The combination of pathway association analysis and the literature search to look for genes already found to be involved in human IBD [18] identified a total of 16 candidate genes potentially associated with IBD in GSD (Table 3). Among these, a total of 11 genes have been previously reported to be associated with human IBD, eight of these genes are also involved in pathways such as Cytokines and Inflammatory Response and Glucocorticoid receptor regulations (Table 3 and S6 Table). Genes encoding for cytokines, including Th2 cytokine, IL-4 and IL-13, were amongst these genes, and were detected by both approaches.

**Table 3:** Enricher results. Genes found to be enriched in biological processes and/or molecular components that are associated or directly/indirectly involved with human IBD.

Chromosme region	gene symbol	gene name	gene start	gene end	database
7	PTPRC*	Protein tyrosine phosphatase, receptor type C	4156687	4282147	KEEG
9	TSC1	Tuberous sclerosis 1	51419149	51454668	KEEG
	RALGDS	Ral guanine nucleotide dissociation stimulator	51308323	51330270	KEEG
	COL5A1	Collagen type V alpha 1 chain	50741552	50856744	Wikipathways20016
	RAPGEF1	Rap guanine nucleotide exchange factor 1	52450597	52562778	Wikipathways20016
11	IL4*	Interleukin-4	20972693	20981541	KEEG; Wikipathways20016; Panther 2016; NCI-Nature 2016
	IL5*	Interleukin-5	20825469	20827269	KEEG; Wikipathways20016; Panther 2016; NCI-Nature 2016
	CSF2*	Granulocyte-macrophage colony-stimulating factor	20344009	20346959	KEEG; Wikipathways20016
	IL13*	Interleukin-13 precursor	20958464	20961391	KEEG; Wikipathways20016; Panther 2016; NCI-Nature 2016
	SLC22A4*	Solute carrier family 22 member 4	20598888	20644639	KEEG
	SLC22A5*	Solute carrier family 22 member 5	20659221	20683073	KEEG
	IRF1*	Interferon regulatory factor 1	20772643	20781207	NCI-Nature 2016
	ACSL6*	acyl-CoA synthetase long-chain family member 6	20223930	20285138	-
	IL3*	Interleukin-3	20330480	20332668	-
	PDLIM4*	PDZ and LIM domain 4	20570752	20587263	-
13	YWHAZ	tyrosine 3-monooxygenase/tryptophan 5-monooxygenase activation protein, zeta	2729503	2763613	Wikipathways20016

*Genes found already to be associated with human IBD.

## 4. Discussion

In the present study, we identified candidate genes on several canine chromosomes that are potentially associated with IBD in GSD. These genes may have an impact on the immune-homeostasis in the canine gut, similar as described for the situation in human IBD.

A number of studies have highlighted the role of immunologic mechanisms and in particular, the involvement of specific cytokine subsets in the pathogenesis of canine IBD. Observations of increased numbers of immunoglobulin-containing cells and T cells in inflamed tissues [1, 29–31], upregulated mucosal and luminal expression of nitric oxide metabolites [32,33], and altered serum concentrations of select acute phase proteins, such as C-reactive protein, which is a marker of inflammation and tissue injuries, in IBD dogs [34,35], support the involvement of impaired immunoregulation in pathogenesis of canine IBD.

Our study results suggest a potential involvement of Th2 cytokines in the pathogenesis of IBD in GSDs, similar as described for ulcerative colitis (UC) in humans. According to the cytokine profile, CD4^+^ T cells can be divided into major subtypes, with Th1 cells producing mainly IL-2, IL-12, IFNg, TNFα, favoring the development of a cell-mediated immune response, whereas Th2 cells mainly produce IL-4, IL-5 and IL-13, which results in the induction of a humoral immune response [36].

In humans, IL-13, produced by specialized cells such as NK T-cells, was identified as an important effector cytokine in UC [37], and its release may impair epithelial barrier function by affecting epithelial apoptosis, tight junctions, and restitution velocity [38]. In addition, lamina propria mononuclear cells (LPMCs) from UC patients have been described to secrete significantly higher amounts of IL-13 and IL-5 than LPMCs from CD patients and healthy controls [37,38]. The IL-13 and IL-5 producing LPMCs bear the NK cell specific markers CD161 and detect CD1d, providing evidence that the release of these cytokines is due to activation of NK T-cells [37]. It was also shown that the NK T-cells isolated from UC patients are cytotoxic for HT-29 epithelial cells, and that this cytotoxicity was enhanced further by addition of IL-13 [37]. In addition, IL-13 signalling through the IL-13α2 receptor (IL-13Rα) induces TGF-β1 production and fibrosis [39]. Many functions of IL-13 are shared with the major Th2 cytokine IL-4. Results of studies performed in a murine model of IBD suggest that in T cell receptor α chain–deficient (TCRα^-/-^) mice, treatment with anti–IL-4 Ab resulted in a decrease of Th2 cytokines, and a concomitant increase of IFN-γ, suggesting that not only NK-T cells, but also CD4^+^ Th2 T cells play a major immunopathological role in the induction of IBD [40]. A similar finding of the potential involvement of IL-4 in canine IBD was confirmed by Jergens [41].

It is worth noticing however, that studies in canine IBD revealed contradictory results regarding the cytokine mRNA expression patterns in intestinal biopsies. German et al. [31] reported a mixed Th1-Th2 cytokine expression in GSDs with small intestinal enteropathy. However, this study included only four dogs diagnosed with IBD. In another study, up-regulation of IL-2 and TNF-α mRNA, two classic Th1 cytokines, in the colonic mucosa of dogs with lymphocytic-plasmacytic colitis (LPC) was reported in dogs with large or small intestinal IBD [42]. Furthermore, no differences in the mRNA expression of IL-2, IL-4, IL-5, IL-6, IL-10, IL-12, IL-18, IFN-γ, TNF-α and TGF-β was detected between healthy dogs or dogs with IBD in more recent studies using real-time q-RT-PCR [43,44]. Finally, Jergens et al. [41] demonstrated expression of a diverse range of pro-inflammatory, anti-inflammatory, and regulatory cytokines in the intestinal mucosa, which differed between dogs with IBD and clinically healthy dogs. Results of these studies suggested at the time, that a predominant Th1- or Th2 cytokine mRNA expression in the inflamed mucosa is not a feature of small- or large- intestinal IBD in dogs [41].

The variations in pattern of cytokine expression observed in these studies might be explained by different methods for mRNA quantification, including semi-quantitative RT-PCR [31,41,42] and real-time RT-PCR [43] techniques, the relatively small number of IBD dogs investigated in each study, and a potential over-representation of GSDs with enteropathies in most studies [31,42,43]. Furthermore, and in contrast to the present study, all of these studies involved a mixture of dog breeds, which may impact on the results obtained and could potentially suggest that different cytokine expression patterns are involved in the pathogenesis of IBD in different breeds. Therefore, to obtain a better insight into pathogenesis of the disease, the current study has focused on one breed and investigated relatively large number of dogs with IBD.

The GWAS data obtained in the present study on GSDs are in agreement with results obtained for UC in humans. Here, IBD is the result of disruption of intestinal immunological homeostasis, and particularly the alteration of the normal balance that the gut maintains between inflammatory and regulatory cytokines [45–47]. Interestingly, cytokine analysis in humans suggests a different pathology of the two main types of IBD. Inflammation associated with Crohn’s disease (CD) is believed to be driven by Th1 cells, producing IFN-ɣ and IL 2, and Th17 cells producing IL-17 and IL-22 [48–52]. In contrast, a Th2 cytokine pattern with IL-4 and IL-5 was found to be important in the pathogenesis of UC [50]. Furthermore, there is evidence that impaired regulation of immune responses by regulatory T cells (Treg) play a part in pathogenesis of human IBD [53]. Recently, these cells also have been characterized in dogs [54], and a decreased number of Treg cells was seen in the peripheral blood [55] and duodenal mucosa of dogs with IBD [56,57]. Whether this observation is a result of IBD, leading to the development of Treg cells that are unable to counteract the inflammatory process in the intestine of dogs with IBD, or a primary result of functional impairment of Tregs in dogs with IBD needs to be studied further.

In conclusion, based on the data provided within the present study, Th2 cytokines may have the potential of causing the epithelial cell damage in GSDs with IBD. Targeted re-sequencing of the genes identified in our study will help to identify causative SNPs and subsequent functional analysis of the causal SNPs may reveal insights into mechanisms involved in pathogenesis of canine IBD.

## Supporting information

S1 Fig Q-Q plots. Quantile–quantile plots a, b and c demonstrate the relationship between observed (y-axis) and expected (x-axis) test statistics.
(DOCX)

S1 Table Logistic association results with no covariates added in the model of the top SNPs, that are below the moderate association threshold but that overlapped with the case-control analysis. (DOCX)

S2 Table Logistic association results of the top SNPs using population structure covariates, that are below the moderate association threshold but that overlapped with the case-control analysis. Note that results are the same as the S1 Table, but the genomic inflation factor is different between the two comparisons.
(DOCX)

S3 Table Single SNP Fst analysis. Here, the top five most divergent SNPS are reported.
(DOCX)

S4 Table Gene list ±1Mb far from the SNPs that are consensus in all the single SNPs analyses. The three consecutive SNP on chromosome 9 were considered as a cluster.
(DOCX)

S5 Table Gene list ±200Kb far from the windows considered for the Fst windows based approach. (DOCX)

S6 Table Detail of the enrichment analyses. Using several databases (KEEG2016. WikiPathways 2016 and NCI-Nature 2016) with Pathway name. P values (P). Adjusted P values (Adj P) and list of genes detected.
(DOCX)
