# Supplementary material for: Genome-wide association studies of inflammatory bowel disease in German shepherd dogs
Source: PLoS One. 2018 Jul 20;13(7):e0200685. doi: 10.1371/journal.pone.0200685 (PMC6054420; doi:10.1371/journal.pone.0200685)
Supplement: S1 Fig — Quantile–quantile plots a, b and c demonstrate the relationship between observed (y-axis) and expected (x-axis) test statistics. (DOCX) [file pone.0200685.s001.docx]

**S1 Fig. Q-Q plots.** Quantile–quantile plots a, b and c demonstrate the relationship between observed (y-axis) and expected (x-axis) test statistics.


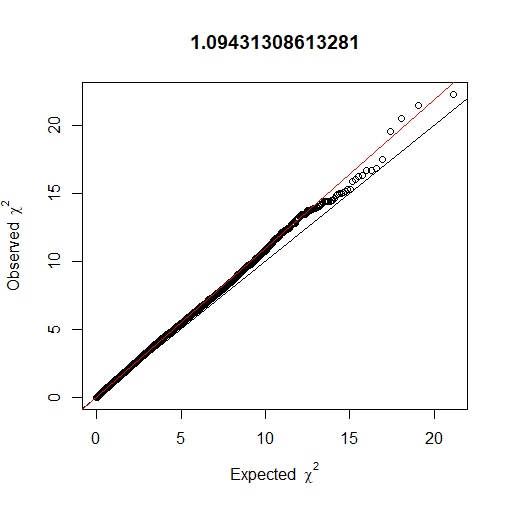

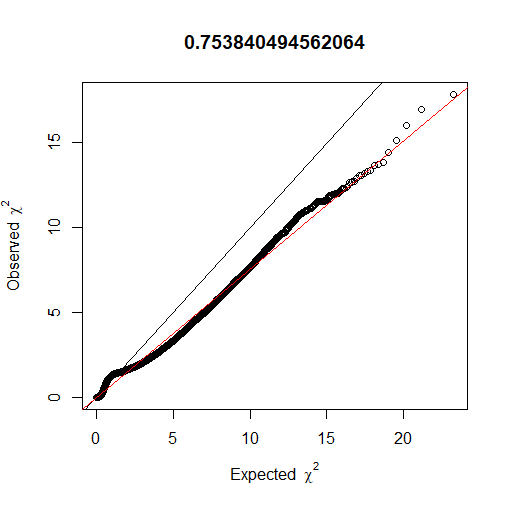


A. Basic association B. Logistic association.


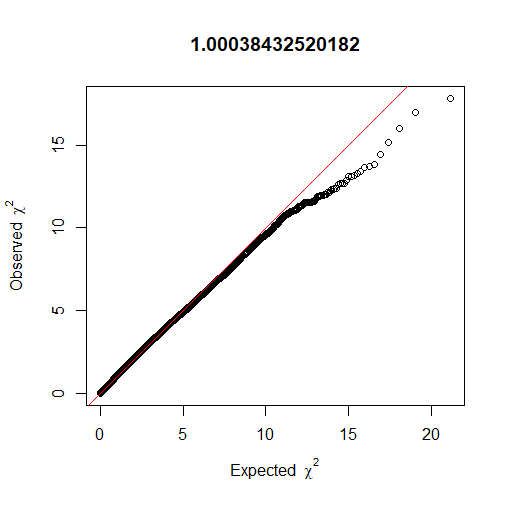


C.Logistic with covariates.
