# Supplementary material for: Genome-wide association studies of inflammatory bowel disease in German shepherd dogs
Source: PLoS One. 2018 Jul 20;13(7):e0200685. doi: 10.1371/journal.pone.0200685 (PMC6054420; doi:10.1371/journal.pone.0200685)
Supplement: S1 Table — (DOCX) [file pone.0200685.s002.docx]

S1 Table: Logistic association results with no covariates added in the model of the top SNPs, that are below the moderate association threshold but that overlapped with the case-control analysis.

| CHR | SNP | BP | A1 | OR | STAT | P |
| --- | --- | --- | --- | --- | --- | --- |
| 9 | BICF2P812982 | 51544743 | G | 0,2383 | -4,223 | 2,41E-05 |
| 9 | BICF2P436494 | 51541093 | G | 0,2472 | -4,118 | 3,82E-05 |
| 9 | BICF2P753594 | 51531181 | C | 0,258 | -4,002 | 6,27E-05 |
| 11 | BICF2S23033111 | 20056580 | C | 4,614 | 3,891 | 1.00E-04 |

CHR: Chromosome

SNP: SNP identifier

BP: Physical position on CanFam3.1

A1: Tested allele (minor allele by default)

OR: odds ratio (--logistic)

STAT: Coefficient t-statistic

P: Asymptotic p-value for t-statistic
