# Supplementary material for: Genome-wide association studies of inflammatory bowel disease in German shepherd dogs
Source: PLoS One. 2018 Jul 20;13(7):e0200685. doi: 10.1371/journal.pone.0200685 (PMC6054420; doi:10.1371/journal.pone.0200685)
Supplement: S3 Table — Here, the top five most divergent SNPS are reported. (DOCX) [file pone.0200685.s004.docx]

S3 Table: Single SNP Fst analyses. Here, the top five most divergent SNPS are reported.

| CHR | SNP | BP | Fst |
| --- | --- | --- | --- |
| 11 | BICF2S23033111 | 20056580 | 0.11 |
| 9 | BICF2P812982 | 51544743 | 0.11 |
| 9 | BICF2P436494 | 51541093 | 0.11 |
| 9 | BICF2P753594 | 51531181 | 0.10 |
| 12 | BICF2S23038091 | 47913517 | 0.09 |

CHR: Chromosome

SNP: SNP identifier

BP: Physical position on CanFam3.1

Fst: Fst value
