# Supplementary material for: Genome-wide association studies of inflammatory bowel disease in German shepherd dogs
Source: PLoS One. 2018 Jul 20;13(7):e0200685. doi: 10.1371/journal.pone.0200685 (PMC6054420; doi:10.1371/journal.pone.0200685)
Supplement: S4 Table — The three consecutive SNP on chromosome 9 were considered as a cluster. (DOCX) [file pone.0200685.s005.docx]

S4 Table: Gene list ±1Mb far from the SNPs that are consensus in all the single SNPs analyses. The three consecutive SNP on chromosome 9 were considered as a cluster

| chr:position | gene start | gene end | gene symbol |
| --- | --- | --- | --- |
| 9:51544743-51531181 | 50525128 | 50575890 | RXRA |
|  | 50741552 | 50856744 | COL5A1 |
|  | 50980055 | 50998116 | OLFM1 |
|  | 51199252 | 51202800 | PPP1R26 |
|  | 51211628 | 51215764 | MRPS2 |
|  | 51274700 | 51284406 | GBGT1 |
|  | 51337183 | 51345182 | CEL |
|  | 51345108 | 51362422 | GTF3C5 |
|  | 51380575 | 51393420 | GFI1B |
|  | 51419149 | 51454668 | TSC1 |
|  | 51461381 | 51465040 | SPACA9 |
|  | 51470756 | 51598690 | AK8 |
|  | 51610139 | 51630655 | GTF3C4 |
|  | 51704590 | 51711197 | BARHL1 |
|  | 51721892 | 51852504 | CFAP77 |
|  | 51853151 | 51878459 | TTF1 |
|  | 51901233 | 51984852 | SETX |
|  | 51994766 | 52059902 | NTNG2 |
|  | 52136577 | 52329198 | MED27 |
|  | 52252792 | 52252917 | RF00092 |
|  | 52450597 | 52562778 | RAPGEF1 |
|  | 52526364 | 52702203 | PRRC2B |
| 11:20056580 | 19532278 | 19532348 | RF01164 |
|  | 19741131 | 19741235 | RF00026 |
|  | 19859680 | 19859981 | RF00100 |
|  | 20094040 | 20094332 | RF00100 |
|  | 20344009 | 20346959 | CSF2 |
|  | 20596496 | 20596603 | RF00026 |
|  | 20825469 | 20827269 | IL5 |
|  | 20950020 | 20950127 | RF00026 |
|  | 20958464 | 20961391 | IL13 |
|  | 20972693 | 20981541 | IL4 |
|  | 19568312 | 19584454 | LYRM7 |
|  | 20330480 | 20332668 | IL3 |
|  | 20512345 | 20548297 | P4HA2 |
|  | 20598888 | 20644639 | SLC22A4 |
|  | 20659221 | 20683073 | SLC22A5 |
|  | 20772643 | 20781207 | IRF1 |
|  | 20988305 | 21043998 | KIF3A |
|  | 21050176 | 21054922 | CCNI2 |
|  | 21052464 | 21079544 | Sept 8 |
|  | 19641735 | 19756030 | CDC42SE2 |
|  | 19982395 | 20134395 | FNIP1 |
|  | 20570752 | 20587263 | PDLIM4 |
