# Supplementary material for: Genome-wide association studies of inflammatory bowel disease in German shepherd dogs
Source: PLoS One. 2018 Jul 20;13(7):e0200685. doi: 10.1371/journal.pone.0200685 (PMC6054420; doi:10.1371/journal.pone.0200685)
Supplement: S5 Table — (DOCX) [file pone.0200685.s006.docx]

S5 Table: Gene list ±200Kb far from the windows considered for the Fst windows based approach.

| CHR | WINDOW START | WINDOW END | gene start | gene end | gene symbol |
| --- | --- | --- | --- | --- | --- |
| 7 | 2500000 | 3500000 | 2304946 | 2305986 | GPR25 |
|  |  |  | 2829878 | 2829980 | RF00026 |
|  |  |  | 2501210 | 2525761 | DDX59 |
|  |  |  | 2548763 | 2602372 | KIF14 |
|  |  |  | 2317285 | 2432636 | CAMSAP2 |
|  |  |  | 2927359 | 3047521 | NR5A2 |
| 7 | 3000000 | 4000000 | 2829878 | 2829980 | RF00026 |
|  |  |  | 3813860 | 3813929 | RF00026 |
|  |  |  | 4055651 | 4055712 | cfa-mir-181b-1 |
|  |  |  | 4055465 | 4055526 | cfa-mir-181a-1 |
|  |  |  | 2927359 | 3047521 | NR5A2 |
|  |  |  | 4156687 | 4282147 | PTPRC |
| 7 | 4000000 | 5000000 | 3813860 | 3813929 | RF00026 |
|  |  |  | 4055651 | 4055712 | cfa-mir-181b-1 |
|  |  |  | 4055465 | 4055526 | cfa-mir-181a-1 |
|  |  |  | 4906182 | 4911746 | C1orf53 |
|  |  |  | 4368429 | 4388551 | ATP6V1G3 |
|  |  |  | 4880215 | 4896928 | LHX9 |
|  |  |  | 4560660 | 4715454 | NEK7 |
|  |  |  | 5139464 | 5261630 | DENND1B |
|  |  |  | 4156687 | 4282147 | PTPRC |
|  |  |  | 3813860 | 3813929 | RF00026 |
|  |  |  | 4055651 | 4055712 | cfa-mir-181b-1 |
|  |  |  | 4055465 | 4055526 | cfa-mir-181a-1 |
|  |  |  | 4368429 | 4388551 | ATP6V1G3 |
|  |  |  | 4560660 | 4715454 | NEK7 |
|  |  |  | 4156687 | 4282147 | PTPRC |
| 13 | 2500000 | 3500000 | 2859051 | 2859163 | RF00019 |
|  |  |  | 2970829 | 2973143 | ZNF706 |
|  |  |  | 3061574 | 3061674 | RF00019 |
|  |  |  | 2381161 | 2394070 | ANKRD46 |
|  |  |  | 2560575 | 2580371 | PABPC1 |
|  |  |  | 2425023 | 2498063 | SNX31 |
|  |  |  | 2729503 | 2763613 | YWHAZ |
|  |  |  | 3204993 | 3363268 | GRHL2 |
|  |  |  | 3366700 | 3447595 | NCALD |
