# Supplementary material for: Genome-wide association studies of inflammatory bowel disease in German shepherd dogs
Source: PLoS One. 2018 Jul 20;13(7):e0200685. doi: 10.1371/journal.pone.0200685 (PMC6054420; doi:10.1371/journal.pone.0200685)
Supplement: S6 Table — Using several databases (KEEG2016. WikiPathways 2016 and NCI-Nature 2016) with Pathway name. P values (P). Adjusted P values (Adj P) and list of genes detected. (DOCX) [file pone.0200685.s007.docx]

S6 Table: Detail of the Enrichment analyses. using several databases (KEEG2016. WikiPathways 2016 and NCI-Nature 2016) with Pathway name. P values (P). Adjusted P values (Adj P) and list of genes detected.

| **Enricher/Pathway** |  |  |  |
| --- | --- | --- | --- |
| **KEEG2016** |  |  |  |
| **Pathway** | P | **Adj P** | Genes involved in the pathway |
| Fc epsilon RI signaling pathway_Homo sapiens_hsa04664 | 0.00004831 | **0.003913** | IL4. IL5. CSF2. IL13 |
| Choline metabolism in cancer_Homo sapiens_hsa05231 | 0.0002253 | **0.005104** | SLC22A4. SLC22A5.TSC1.RALGDS |
| T cell receptor signaling pathway_Homo sapiens_hsa04660 | 0.001056 | **0.02719** | IL4.IL5.CSF2.PTPRC |
| Asthma_Homo sapiens_hsa05310 | 0.0001034 | **0.004186** | IL4.IL5.IL13 |
| Inflammatory bowel disease (IBD)_Homo sapiens_hsa05321 | 0.002904 | **0.05982** | IL4. IL5. IL13 |
| Jak-STAT signaling pathway_Homo sapiens_hsa04630 | 0.004643 | **0.07971** | IL4. IL5.IL13. CSF2 |
|  |  |  |  |
|  |  |  |  |
| **WikiPathways 2016** |  |  |  |
| **Pathway** | P value | **Adjusted p value** | Genes involved in the pathway |
| Cytokines and Inflammatory Response_Homo sapiens_WP530 | 9.73E-07 | **0.00004426** | IL4. IL5. CSF2. IL13 |
| Cytokines and Inflammatory Response (BioCarta)_Mus musculus_WP222 | 8.25E-07 | **0.00004426** | IL4. IL5. CSF2. IL13 |
| Allograft Rejection_Homo sapiens_WP2328 | 0.00008278 | **0.002511** | IL4.IL5. COL5A1. IL13 |
| IL-5 Signaling Pathway_Mus musculus_WP151 | 0.001066 | **0.02426** | IL5. RAPGEF1. YWHAS |
| IL-3 Signaling Pathway_Mus musculus_WP373 | 0.002953 | **0.04588** | RXA. RAPGEF1. YWHAS |
| Inflammatory Response Pathway_Mus musculus_WP458 | 0.003529 | **0.04588** | IL4. IL5 |
| Inflammatory Response Pathway_Homo sapiens_WP453 | 0.003529 | **0.04588** | IL4. IL5 |
|  |  |  |  |
|  |  |  |  |
| **NCI-Nature 2016** |  |  |  |
| **Pathway** | P value | **Adjusted p value** | Genes involved in the pathway |
| Glucocorticoid receptor regulatory network_Homo sapiens_dfba0dfb-6192-11e5-8ac5-06603eb7f303 | 4.309E-06 | **0.0002543** | IL4. IL5. CSF2. IL13. IRF1 |
| Calcineurin-regulated NFAT-dependent transcription in lymphocytes_Homo sapiens_0439e9da-618f-11e5-8ac5-06603eb7f303 | 0.0003382 | **0.009978** | IL4. IL5. CSF2 |
| AP-1 transcription factor network_Homo sapiens_3ce2f9c5-6189-11e5-8ac5-06603eb7f303 | 0.001113 | **0.02188** | IL4. IL5. CSF2 |
| Calcium signaling in the CD4+ TCR pathway_Homo sapiens_5294f70b-618f-11e5-8ac5-06603eb7f303 | 0.0033 | **0.0473** | IL4.CSF2 |
|  |  |  |  |
| GMCSF-mediated signaling events_Homo sapiens_095aa3ef-6193-11e5-8ac5-06603eb7f303 | 0.005053 | **0.04969** | CSF2. YWHAS |
| IL12 signaling mediated by STAT4_Homo sapiens_72cf19b8-6193-11e5-8ac5-06603eb7f303 | 0.004009 | **0.0473** | IRF1.IL13 |
